# Erector spinae plane block

**DOI:** 10.1016/j.bjae.2026.06.004

**Published:** 2026-07-10

**Authors:** A. De Cassai, S. Coppens, B. Dost, K. El-Boghdadly

**Affiliations:** 1 University of Padua and University Hospital of Padua, Padua, Italy; 2 University of Leuven and University Hospitals Leuven, Leuven, Belgium; 3 Ondokuz Mayis University, Samsun, Türkiye; 4 Guy’s and St Thomas’ NHS Foundation Trust, London, UK; 5 King’s College London, London, UK

**Keywords:** anaesthesia, analgesia, conduction, interventional, nerve block, pain, postoperative, paraspinal muscles, ultrasonography

**Key points**

• Spread of local anaesthetic to the paravertebral space is inconsistent and unreliable with erector spinae plane block (ESPB).
• The strongest clinical evidence currently supports using ESPB in major spinal surgery, while results in other surgical settings are more modest and heterogeneous.
• Local anaesthetic doses should be individualised, as large volumes may increase the risk of local anaesthetic systemic toxicity.

**Learning objectives**

By reading this article, you should be able to:

• Identify the key anatomical structures and ultrasound landmarks relevant to the erector spinae plane block (ESPB).
• Summarise the proposed mechanisms of action of the ESPB.
• Describe the practical steps for performing ESPB, including positioning of the patient, needle placement and local anaesthetic dosing.
• Discuss the clinical indications for ESPB and recognise its contraindications and potential complications.

The erector spinae plane block (ESPB) was first described in 2016 as an interfascial plane technique for the management of thoracic neuropathic pain.^1^ It was rapidly embraced by the scientific and clinical communities and, over the last 10 yr, it has been proposed for indications such as perioperative analgesia for thoracic, breast, abdominal, spinal and orthopaedic surgery, as well as for patients with trauma and rib fractures. The appeal of ESPB lies in its apparent technical simplicity, ultrasound-guided reproducibility and perceived safety profile. Collectively, these features have led to its inclusion among the so-called ‘Plan A’ blocks, regarded as foundational regional anaesthesia techniques with which all anaesthetists should be familiar, even without advanced expertise in regional anaesthesia.^2^

ESPB block was first described a decade ago and has been widely adopted, sometimes with enthusiasm not justified by the supporting body of evidence. In this article we review the relevant anatomy, sonoanatomy, technique, proposed mechanisms of action, indications and contraindications, as well as potential complications associated with the ESPB.

## Relevant anatomy and sonoanatomy

**Fig 1:**
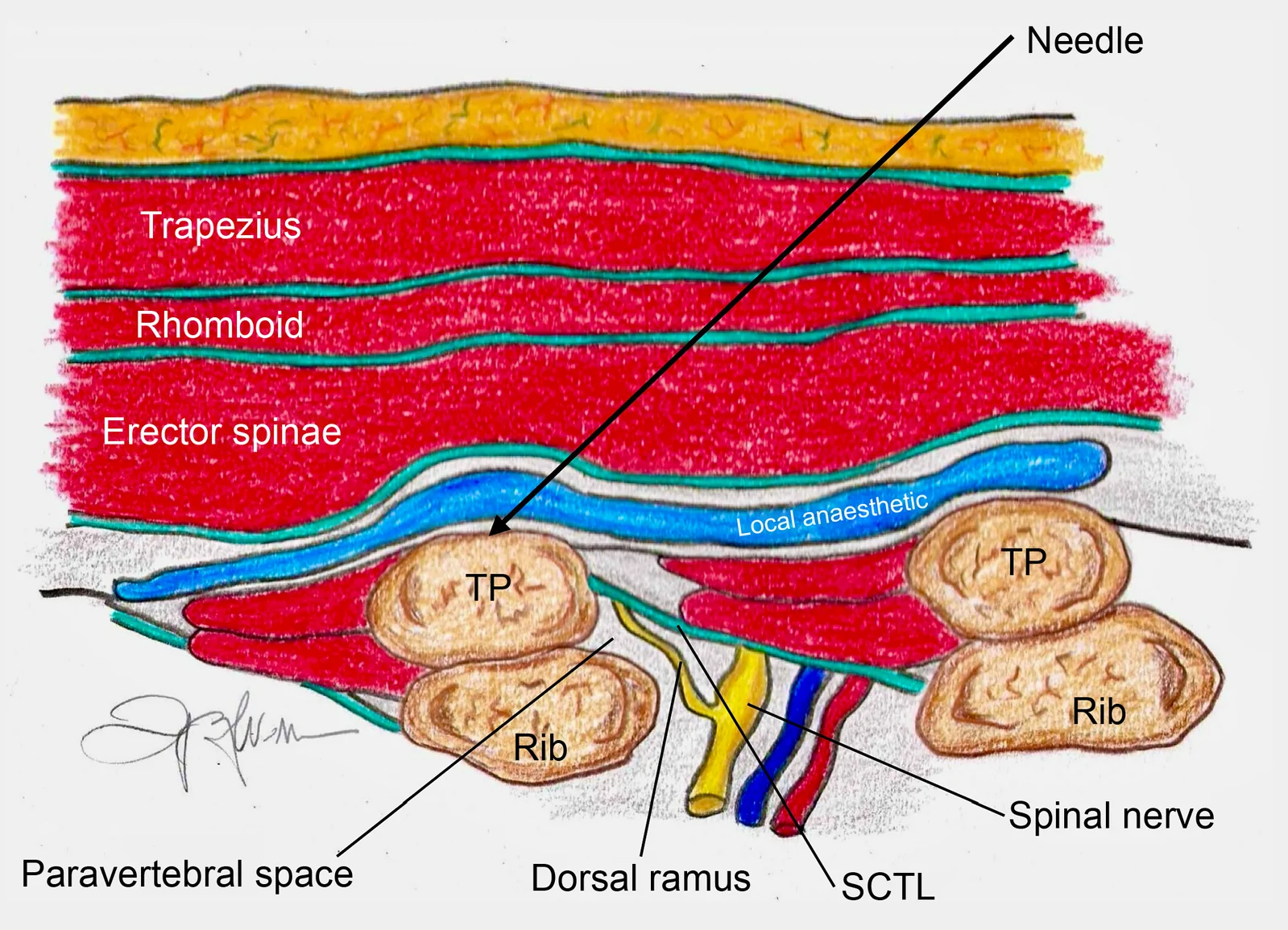
Schematic representation of needle placement and injectate spread during erector spinae plane block. The needle is advanced in-plane until contact with the transverse process is achieved. Local anaesthetic is then injected deep to the erector spinae muscle, separating it from the transverse process and spreading in a cranio-caudal direction within the erector spinae plane.

The ESPB targets a potential fascial plane located deep to the erector spinae muscle group and superficial to the transverse processes of the vertebrae (Fig. 1).

The erector spinae muscle group comprises three longitudinal columns: iliocostalis (lateral), longissimus (intermediate) and spinalis (medial). These muscles share a broad common tendinous origin arising from a thick aponeurotic sheet of the thoracolumbar fascia attached to the posterior iliac crest, posterior sacrum, sacroiliac ligaments and the spinous processes of the lower lumbar vertebrae. From this common origin, the muscles ascend cranially and diverge: the iliocostalis inserts onto the angles of the ribs and the transverse processes of the lower cervical vertebrae; the longissimus inserts onto the transverse processes of the thoracic and cervical vertebrae and, superiorly, onto the mastoid process; and the spinalis inserts onto the spinous processes of the upper thoracic and cervical vertebrae, forming the most medial column of the group.^3^

This plane extends longitudinally along the posterior thoracic and abdominal wall and is bounded anteriorly by the transverse processes and superior costotransverse ligaments and posteriorly by the dense investing fascia of the erector spinae muscle group.

Importantly, the erector spinae plane is not a true anatomical compartment or a classical fascial plane, as it is not a defined potential space bounded between two distinct fascial layers but a space whose capacity to accommodate injectate depends on several factors. These include tissue compliance, injection volume, injection dynamics and vertebral level. However, numerous factors, many of which are incompletely understood, mean that spread within this anatomical region remains largely unpredictable and not entirely reproducible.^4^

At the thoracic level, the dorsal rami emerge posteriorly from spinal nerves, traverse the paraspinal musculature and supply the posterior thoracic wall, whereas the ventral rami course anteriorly within the paravertebral space to form the intercostal nerves. These two neural pathways are separated by osseous and ligamentous structures, including the transverse processes and costotransverse ligaments, and no consistent anatomical conduit exists between the erector spinae plane and the paravertebral or epidural spaces. This anatomical separation is fundamental for understanding the strengths and limitations of ESPB. It also provides, at least in part, an explanation for the mixed results from cadaveric studies trying to demonstrate dye staining at paravertebral or even anterior rami levels.^5^, ^6^, ^7^

**Fig 2:**
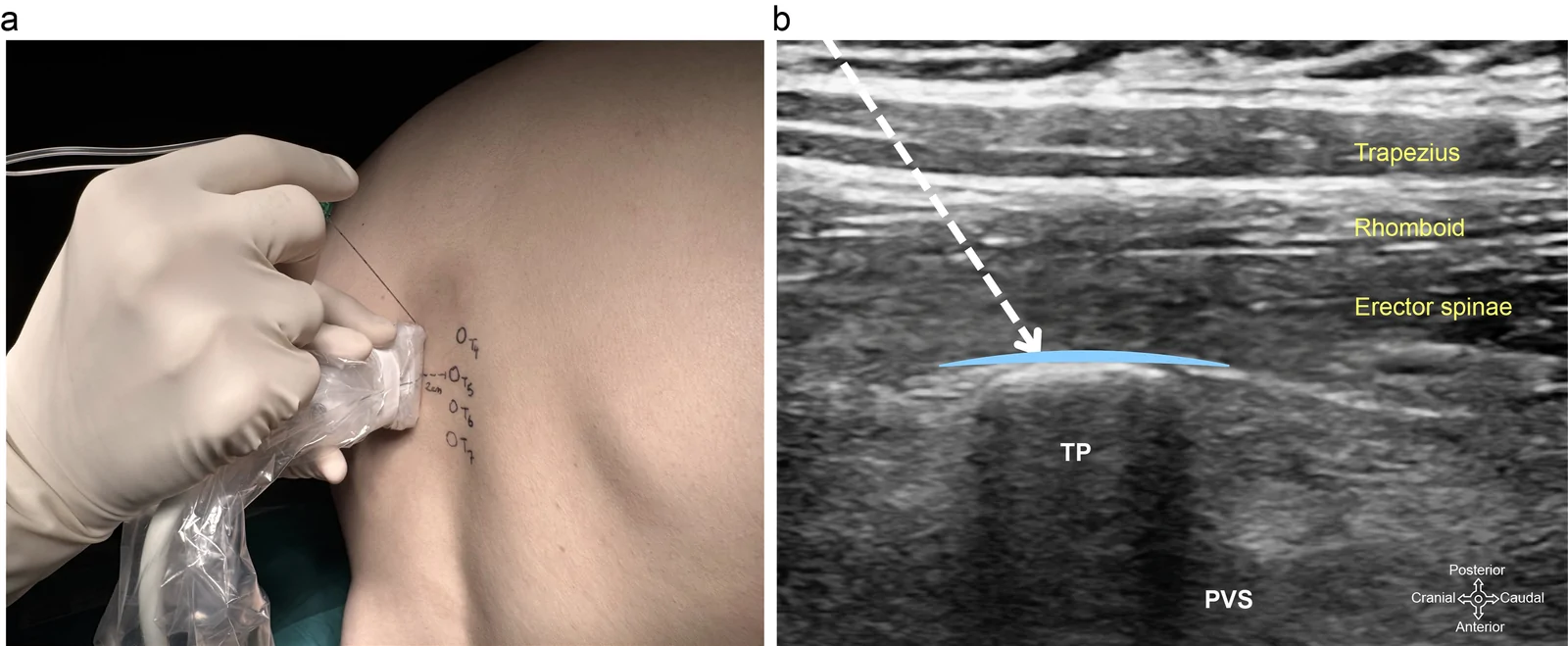
Probe placement and corresponding sonoanatomy. (A) Positioning the patient and probe placement for the ESPB, with the ultrasound transducer oriented in a longitudinal parasagittal plane over the transverse processes. (B) Corresponding ultrasound image demonstrating relevant anatomy: trapezius, rhomboid major, and erector spinae muscles superficial to the transverse process, with the paravertebral space located deep and anterior. The needle is advanced in-plane in a cranial-to-caudal direction to contact the transverse process, and local anaesthetic is deposited deep to the erector spinae muscle, resulting in separation of the muscle from the transverse process (hydrodissection). ESPB, erector spinae plane block.

**Fig 3:**
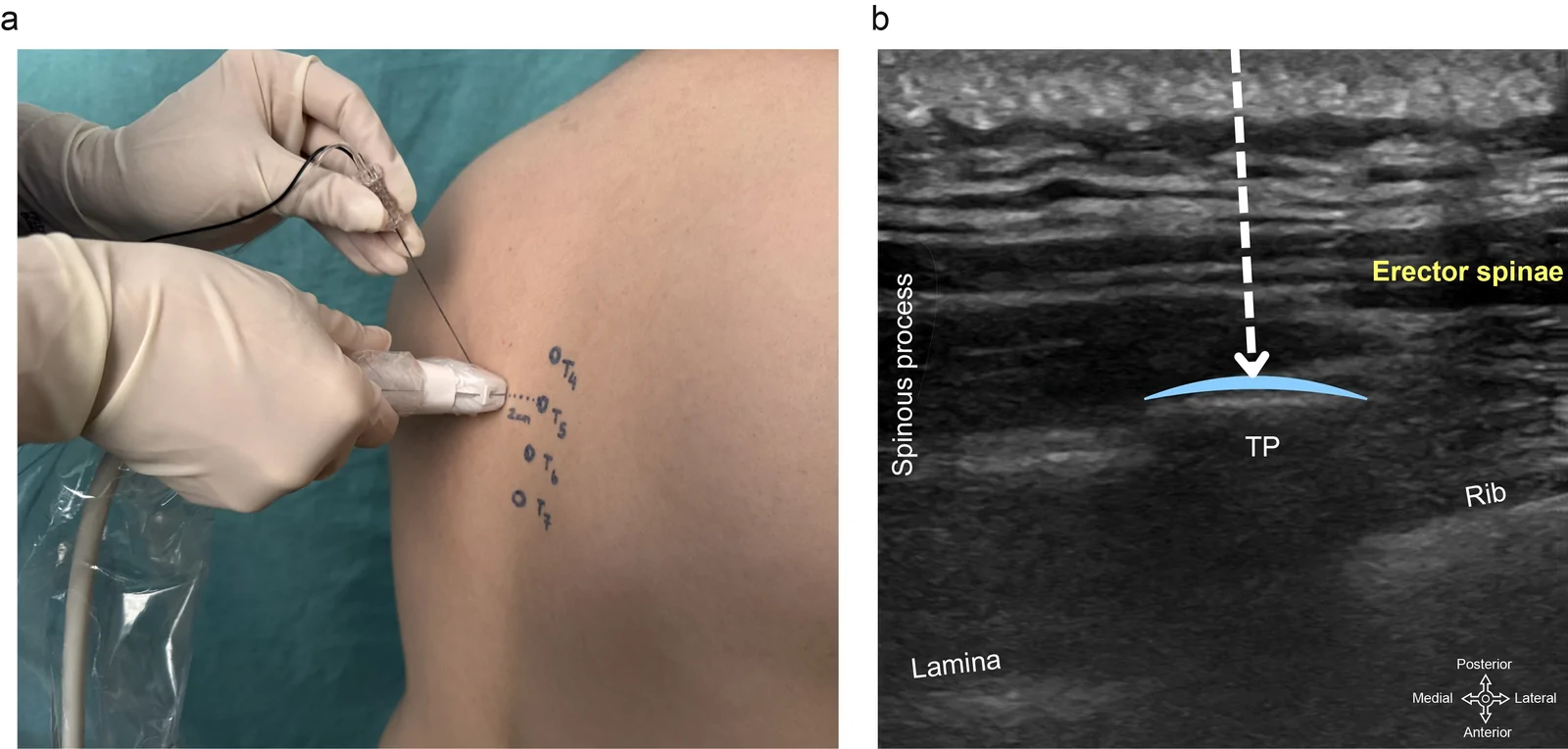
Probe placement and corresponding sonoanatomy for the transverse approach to ESPB. (A) Positioning the patient and ultrasound probe placement for the ESPB, with the transducer oriented in a transverse plane at the thoracic level, lateral to the spinous processes. (B) Corresponding ultrasound image demonstrating the relevant sonoanatomy. The transverse process (TP) is identified deep to the erector spinae muscle. Adjacent structures including the spinous process, lamina, and rib are also visible. The needle is advanced toward the transverse process, and local anaesthetic is deposited deep to the erector spinae muscle, producing separation of the muscle from the transverse process (hydrodissection). ESPB, erector spinae plane block.

From a sonoanatomical perspective, ESPB is most performed using a parasagittal longitudinal ultrasound approach (Fig. 2), but a transverse approach is also possible (Fig. 3).

At the upper thoracic levels, above the fifth thoracic vertebral level (T5), the trapezius and rhomboid major muscles may be visualised superficial to the erector spinae muscle group, whereas at mid- and lower thoracic levels, the erector spinae muscles lie directly beneath the subcutaneous tissues.^8^

**Fig 4:**
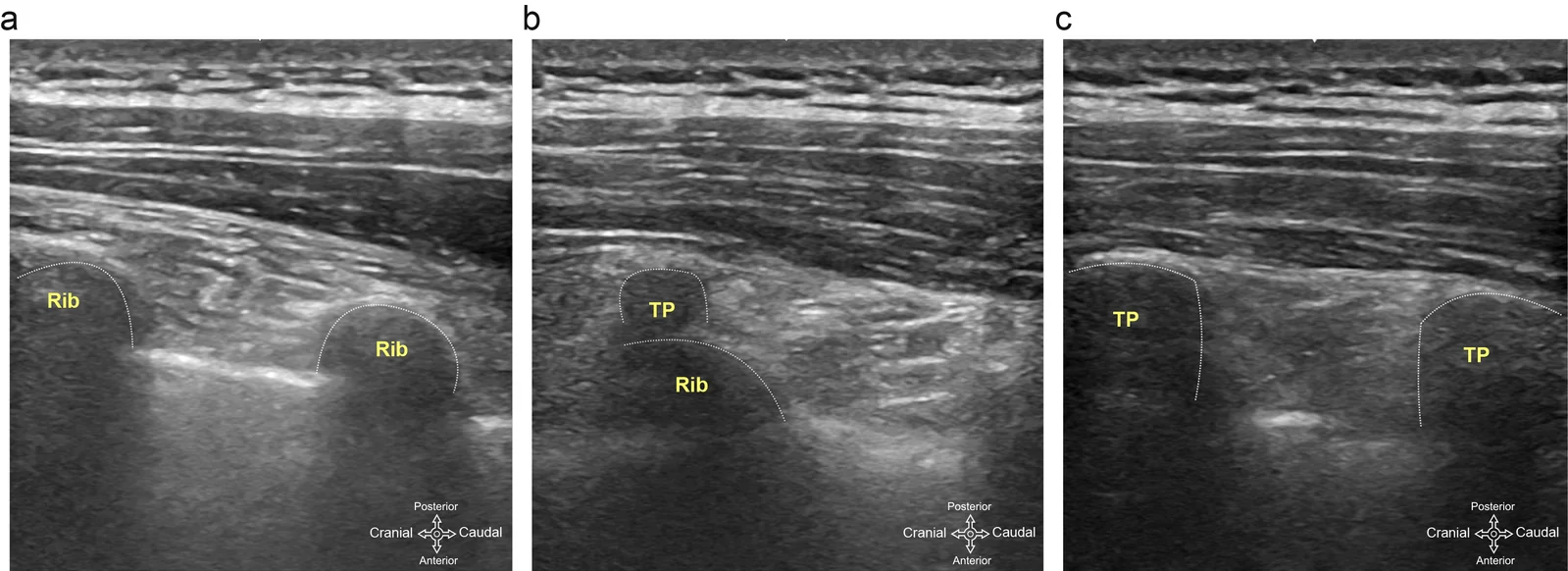
Sonoanatomy from rib to transverse process for ESPB. Sequential ultrasound images demonstrating probe translation from lateral to medial: (A) Rib view, showing the rounded acoustic shadow of the rib. (B) Transition at the costotransverse junction, where the rib and transverse process are both visualised. (C) Transverse process view, identified by its flatter, squared acoustic shadow, providing the target for ESPB needle placement. ESPB, erector spinae plane block. ESPB, erector spinae plane block.

From superficial to deep, the key structures include the skin, subcutaneous tissue and erector spinae muscle group. Deep to the erector spinae muscle group, the posterior elements of the vertebrae and, at thoracic levels, the adjacent ribs generate a characteristic hyperechoic line with posterior acoustic shadowing, which serves as a reliable sonographic end point for needle advancement. Several osseous structures may be visualised at this depth and can be distinguished based on their shape, orientation, and relationship to surrounding landmarks. The ribs appear as rounded hyperechoic structures with posterior acoustic shadowing and are typically identified more laterally. The transverse processes appear as more rectangular or squared hyperechoic structures with a flat acoustic shadow and are located medial to the ribs. At the rib–transverse process junction, corresponding to the costotransverse angle, the transverse process typically lies more superficial than the rib. This transition can be appreciated during probe movement and may aid in recognising the relevant anatomy (Fig. 4). Medially, the lamina appears as a continuous, flatter hyperechoic line extending towards the spinous process and often forms a gently sloping bony contour. The spinous process is visualised in the midline as a prominent hyperechoic structure associated with a dense posterior acoustic shadow.

The pleura lies deeper and anterior to the transverse process and may be visualised as a hyperechoic line exhibiting rhythmic movement synchronous with respiration, representing lung sliding.

## Mechanisms of action

Although the ESPB has gained considerable attention partly for its relative technical simplicity, its mechanism of action is far from simple. In fact, it is unlikely to be explained solely by local anaesthetic spread within a single plane; rather, it probably results from a combination of anatomically plausible and mechanistically complementary pathways.

The most consistent and reproducible mechanism is blockade of the dorsal rami and their posterior cutaneous branches as local anaesthetic spreads within the erector spinae plane. This results in reliable analgesia of the posterior thoracic wall and paraspinal musculature, and aligns closely with both the injection site and the observed sensory distribution in many patients.

**Fig 5:**
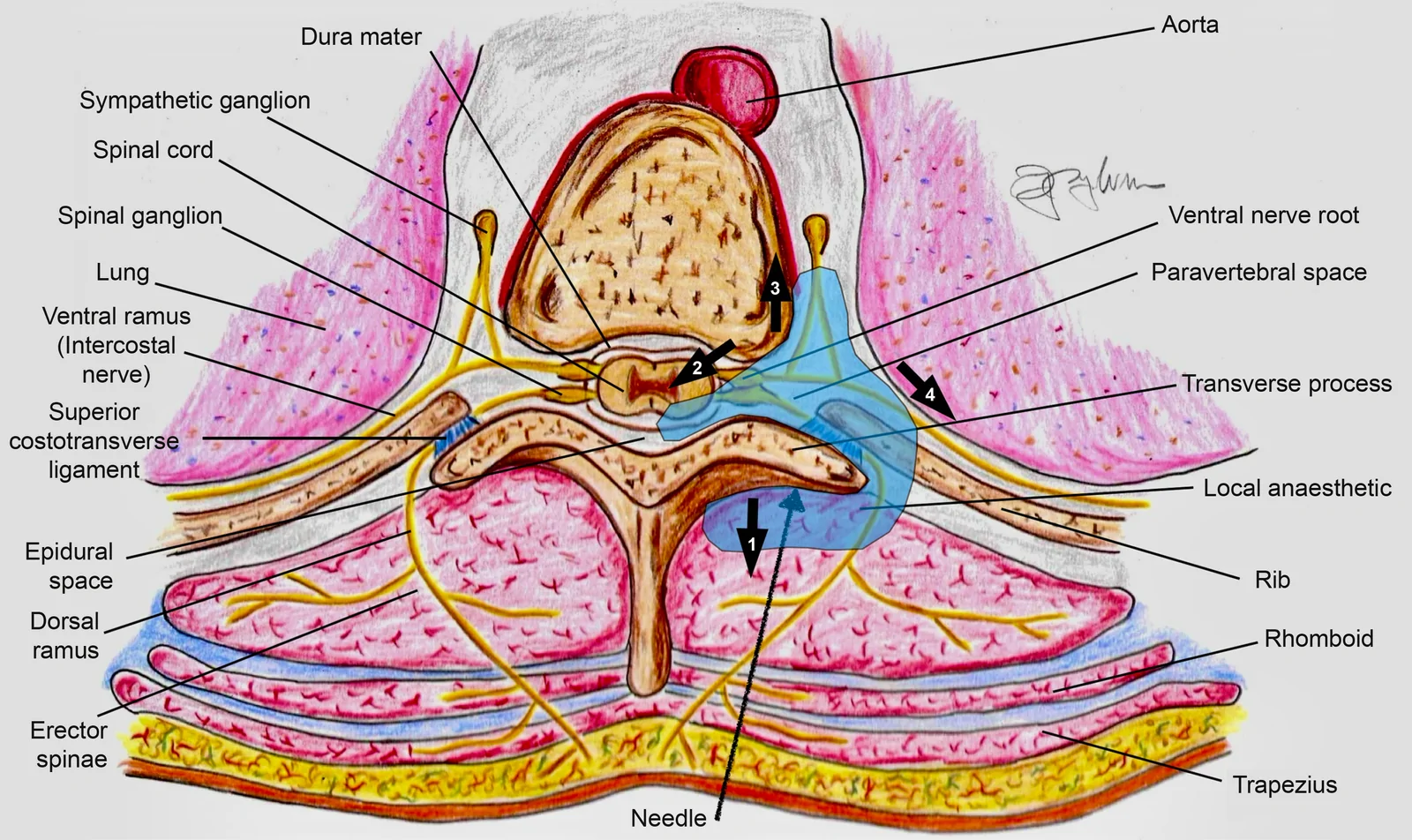
Transverse schematic of the ESPB and proposed mechanisms of action. Local anaesthetic is deposited deep to the erector spinae muscle and superficial to the transverse process. The most consistent effect is blockade of the dorsal rami within the erector spinae plane (1). Anterior spread toward the paravertebral space (2) may occur but appears variable and inconsistent, with possible involvement of the ventral nerve root, ventral ramus (intercostal nerve), and sympathetic ganglion. Rare epidural extension (3) and lateral spread toward the intercostal region (4) have also been described. ESPB, erector spinae plane block.

The spread of local anaesthetic into the paravertebral space, with potential involvement of the ventral rami and sympathetic chain, has been demonstrated in cadaveric, radiological and volunteer studies.^9^, ^10^, ^11^ However, as previously suggested, such spread appears inconsistent and dependent on injectate volume, injectate dynamics, vertebral level and individual anatomy. The spread is likely to occur through discontinuities in connective tissue or along vascular and ligamentous pathways, rather than through a predictable anatomical channel. For this reason, paravertebral spread should be regarded as possible rather than fundamental to the mechanism of ESPB, and the block should not be considered a reliable surrogate for thoracic paravertebral blockade, particularly given that paravertebral spread has been reported to occur in 57% of cases, with similar proportions observed across studies involving both cadavers and volunteers.^12^ However, it should be recognised that cadaveric studies, frequently used as a surrogate to evaluate injectate spread in fascial plane blocks, have inherent limitations, including alterations in tissue properties after death, differences between dye and local anaesthetic behaviour, and the potential for tissue disruption or contamination during dissection.^13^ Epidural spread has also been reported but remains rare and is neither necessary nor sufficient to explain the analgesic effects observed clinically (Fig. 5).^11^

However, spread of injectate in ESPB is also likely to be dynamic. Spread is influenced by several factors related to physiology and performance of the procedure, including respiratory movement; gravity; positioning of the patient; and injected volume, pressure and direction.^4^ These factors may contribute to the marked variability in spread patterns observed between studies and limit the ability to define a single ‘ideal’ injection end point.

Given the relatively large volumes of local anaesthetic drugs commonly used for ESPB, systemic absorption may contribute modestly to analgesia.^14^ Pharmacokinetic studies of ESPB have demonstrated rapid systemic uptake, sometimes approaching or exceeding plasma concentrations associated with intravenous lidocaine infusions, especially when high volumes are used and vasoconstrictors are omitted.^15^ However, systemic absorption produces diffuse analgesia rather than dermatomal or segmental sensory blockade, and therefore cannot be considered the primary mechanism of action.

Increasing attention has focused on neuromodulatory and immunomodulatory mechanisms to explain the clinical effects of the ESPB, particularly modulation of nociceptive input at the level of the dorsal horn and within richly innervated paraspinal tissues.^16^ Deep paraspinal tissue is now recognised as a biologically active structure containing dense networks of nociceptive, autonomic and immune-responsive fibres, as demonstrated in detail in recent anatomical and histological studies.^17^^,^^18^

Local anaesthetic deposition within the erector spinae plane may therefore reduce afferent input from the dorsal rami and nociceptors, with secondary attenuation of dorsal horn excitability and central sensitisation. From this perspective, ESPB is best conceptualised as a posterior thoracic and muscular analgesic technique that modulates nociceptive transmission rather than producing predictable dense segmental anaesthesia through ventral ramus or sympathetic blockade.

Understanding these mechanisms is essential when interpreting the heterogeneous results reported in the ESPB literature. Variability in clinical efficacy is likely to reflect anatomical and mechanistic diversity rather than technical failure; unrealistic expectations should be avoided, particularly regarding consistent ventral ramus or sympathetic blockade. When used with an appreciation of its mechanistic limitations, ESPB remains a possible component of multimodal opioid-sparing perioperative analgesia for certain indications.

## Technique

### Positioning and selecting the vertebral level

The ESPB is usually performed with the patient in the sitting position; however, it can also be performed in the lateral or prone position when required, for example, when the block is placed after induction of anaesthesia. The optimal position allows a stable ultrasound image and precise needle control. The selection of the vertebral level should be guided by the surgical incision and the expected pain sites to align the transverse process with the dominant dermatomes.

### Probe placement

A high-frequency linear probe is generally suitable for slim patients at the upper thoracic level. However, at the lower thoracic and lumbar levels, the transverse process may lie relatively deep because of the overlying muscle layers, in which case a low-frequency curvilinear probe provides superior penetration and improved contrast for visualising the relevant tissue planes. The probe is placed in a longitudinal parasagittal orientation to identify the key anatomical landmark previously described and, in particular, the transverse process, which appears as a flat hyperechoic line with posterior acoustic shadowing. Mistaking the rib for the transverse process is a common error that may contribute to an ineffective block; sequential identification of the rib, transverse process and lamina can improve anatomical orientation and procedural accuracy. In particular, practitioners should actively identify the rib–transverse process transition, which serves as a valuable landmark for the reliable differentiation of these structures.

### Needle approach and target plane

An in-plane needle approach is generally preferred, as it allows continuous visualisation of the needle shaft and tip. A 22–25 gauge needle with a length of approximately 50–80 mm at the thoracic level and 80–100 mm at the lumbar level is commonly used, depending on patient habitus and target depth. The needle may be advanced in either the craniocaudal or caudo-cranial direction until bony contact with the transverse process is achieved, which serves as a reliable depth marker. The needle is then withdrawn by approximately 1–2 mm to position the tip beneath the deep surface of the erector spinae muscle.

A small test injection should be given to confirm the correct needle placement. The key sonographic indicator of successful positioning is visible hydrodissection, characterised by the separation of the erector spinae muscle from the transverse process and longitudinal craniocaudal spread of the injectate along the plane. The injections often cover multiple vertebral levels, which explains the frequent observation of multisegmental posterior thoracic analgesia in clinical practice.

The needle endpoint is typically described as the transverse process. However, it has been suggested that injecting local anaesthetic between the transverse processes may result in greater anterior spread compared with injections targeting the medial aspect of the transverse process. Readers should be aware, but, that any injection performed deeper than the erector spinae plane is more consistent with an intertransverse process block rather than an ESPB; although these techniques share similarities, they differ in patterns of spread and clinical effects.^19^

In this context, Lee and colleagues^20^ proposed the use of electrical nerve stimulation through the block needle as an adjunct to ultrasound guidance to help confirm needle placement during ESPB (use of electrical stimulation to confirm the erector spinae plane). In their report, electrical stimulation produced segmental paraspinal muscle contractions, supporting the location of the needle within the erector spinae muscle compartment rather than in deeper paravertebral structures. We suggest that this technique may help differentiate superficial placement within the erector spinae plane from deeper needle advancement towards the intertransverse or paravertebral spaces. Although this approach requires further validation, it provides a potentially useful method to better characterise needle tip position and may contribute to clarifying the ongoing debate regarding the precise anatomical target of ESPB.

In adults, a typical volume of 15–30 ml of a long-acting local anaesthetic such as ropivacaine, bupivacaine or levobupivacaine is used. In children, weight-based dosing of approximately 0.1–0.3 ml kg^-1^ is commonly used. The local anaesthetic concentration should be selected in conjunction with the total volume and maximum recommended dose per kilogram. Typical concentrations include ropivacaine 0.2–0.5% and bupivacaine 0.25–0.375%. The erector spinae plane is characterised by rapid adsorption. Thus, the selection of local anaesthetic drug and volume should be individualised according to the clinical context, including unilateral or bilateral application, the patient’s weight, and comorbidities, without using ‘standard’ dosing.^15^ Although the ESPB is often regarded as a ‘high-volume’ technique, increasing the volume should not be used to compensate for incorrect needle placement; injection resulting in localised muscle swelling without plane separation suggests intramuscular placement. In such cases, further injection should be avoided, and the needle should be repositioned until the correct plane is identified.

A transverse approach is also possible. In this case, the transducer is positioned in the transverse plane at the intended block level with the spinous process centred on the ultrasound screen and then gently slid laterally (Fig. 3). From this perspective, the laminae and transverse processes can be identified beneath the overlying paraspinal muscles. The block can be performed using either an in-plane lateral-to-medial approach or an out-of-plane technique. The transverse approach can be applied at both the thoracic and lumbar levels. At the L3–4 level, lumbar ESPB may also be performed using the ultrasound view obtained when performing the lumbar plexus block method known as the Shamrock technique.^21^

The sacral ESPB represents a caudal extension of the erector spinae plane concept to the sacral region. Under ultrasound guidance, local anaesthetic is deposited deep to the sacral multifidus muscle and superficial to the dorsal sacral surface, using either a paramedian or a midline approach. Cadaveric and imaging studies suggest craniocaudal spread with variable involvement of the dorsal sacral rami and, in selected cases, limited anterior spread through the sacral foramina.^22^

### Continuous erector spinae plane catheter

Continuous erector spinae plane catheters may be considered when the need for prolonged analgesia is expected, such as after thoracic surgery, rib fractures, complex spinal surgery or selected major procedures in which repeated boluses or infusions may reduce opioid requirements.^23^ However, catheter placement should be viewed as an advanced technique rather than routine practice. The continuous technique may be performed using a catheter-through-the-needle approach with a Tuohy needle or a catheter-over-the-needle system.^24^

Continuous ESPB may be limited by catheter migration or loss of accurate placement within the target plane. The potential for migration within a relatively loose compartment highlights the importance of careful technique, secure catheter fixation and clinical monitoring of block effectiveness when continuous ESPB is used.^25^

The target plane for catheter insertion is identical to that of the single-shot technique. After confirming correct hydrodissection, the catheter is advanced within the plane under ultrasound guidance. Secure fixation is essential because catheter migration and dislodgement are common practical challenges. Infusion strategies must be planned carefully to avoid excessive cumulative local anaesthetic dosing, particularly when bilateral catheters are used.

Despite the theoretical advantages of programmed intermittent bolus techniques for continuous erector spinae plane catheters, current evidence has not demonstrated clear superiority over continuous infusion in terms of patient-centred outcomes, particularly in the context of modern infusion pumps and robust multimodal analgesia regimens. The variability in injectate spread, uncertainties regarding optimal dosing and differences in catheter performance may partially explain these results. Consequently, optimisation of dosing patterns and a better understanding of injectate distribution remain important areas for future research.^26^

## Indications

The ESPB has been used for a wide range of surgical and non-surgical analgesic indications (Table 1) and has been proposed in numerous guidelines, although the quality of published evidence is generally limited.^27^

**Table 1:** Indications, evidence and alternatives to the erector spinae plane block. TAP, transversus abdominis plane.

Surgical site	Evidence base	Limitations	Alternative
Thoracic surgery	Limited	Not better than alternatives	Paravertebral block likely to be more beneficial
Breast surgery	Limited	Analgesia only in the first 2 h after surgery	Paravertebral or interpectoral plane blocks likely to be more beneficial
Spinal surgery	Modest	Technically difficult if performed after surgery	Likely to be more beneficial and technically easier because of the surgical setting than epidural
Abdominal surgery	Modest	Technically difficult to position compared with alternatives	TAP block likely to be more beneficial and technically easier
Caesarean section	Limited	Technically difficult to position compared with alternatives, only if intrathecal morphine not used	TAP block likely to be more beneficial and technically easier
Rib fractures	Modest	Failure rate	Likely to be more beneficial than epidural and paravertebral block

### Truncal surgery

Evidence suggests that the ESPB has modest or variable clinical benefit in thoracic surgery compared with a control, paravertebral block, intercostal nerve block or serratus anterior plane blocks.^28^ Although some data suggest that Quality of Recovery-15 scores may be improved compared with paravertebral block, particularly in the psychological and emotional states, these improvements do not appear to result from analgesic effectiveness and are not consistently demonstrated.^29^ Given the limited clinical benefit, ESPB for thoracic surgery might be best reserved for a cohort of patients in whom alternative techniques, including thoracic epidural and paravertebral block, might not be suitable, as per PROSPECT guidance.^30^

In breast surgery, data suggest that the ESPB also has modest analgesic effectiveness, but most of this benefit is in the early postoperative period.^31^ It appears to be less effective than paravertebral block and interpectoral plane blocks in several analgesia-related outcomes.^32^ Thus, similar to thoracic surgery, the ESPB might have a more limited role for the majority of patients if interpectoral, serratus anterior or paravertebral blocks are possible.

### Spinal surgery

The data supporting the use of ESPB in major spinal surgery are more favourable. Compared with no block, ESPB provides a demonstrable analgesia effect in terms of pain scores, opioid consumption and quality of recovery. Whilst other regional anaesthesia techniques may also be effective, the ESPB appears to be the most widely implemented for this type of surgery. This is a particularly strong indication for the ESPB, particularly in patients with a high risk of moderate or severe postoperative pain.^33^

### Abdominal surgery

Many different regional anaesthesia techniques can be used for different abdominal surgical procedures, including ESPB. Data suggest that the analgesic benefits are greater with ESPB than systemic analgesia alone, but are similar to the quadratus lumborum block and transversus abdominis plane (TAP) block.^34^ Given the ease of access to the relevant anatomy in the supine position for TAP and quadratus lumborum blocks, compared with the more challenging access to the erector spinae plane in the lower thoracic and lumbar region, the former are generally preferred in the majority of patients over ESPB.

### Caesarean section

In the presence of intrathecal morphine, there is no evidence that any fascial plane block adds meaningful analgesic benefit.^35^ Although previous PROSPECT guidelines recommended fascial plane blocks in the absence of intrathecal morphine, recent evidence demonstrates that the quadratus lumborum block was more effective than erector spinae plane and TAP blocks.^36^ Given the ease of access to the relevant anatomy for a TAP block, this is preferred in most patients over an ESPB.

### Rib fractures

Thoracic epidural analgesia has traditionally been used for rib fractures, but this technique is technically complex and associated with concerns regarding haemodynamic and coagulation-related challenges. Alternatives included the paravertebral block, serratus anterior plane block and intercostal nerve blocks. Although evidence does not clearly distinguish the ESPB from other fascial plane techniques, the ESPB is likely to be more useful, given the relative ease of insertion compared with thoracic epidurals and paravertebral blocks, whilst also being potentially suitable for posterior and anterolateral rib fractures.^37^

## Contraindications

As with most procedures, absolute contraindications include the patient’s refusal, allergy to any of the drugs or products used and local infection at or near the site of catheter insertion. According to the joint European Society of Anesthesiology and Intensive Care and European Society of Regional Anaesthesia coagulation guidelines in patients receiving regional anaesthesia, ESPB is classified as a superficial block and is therefore associated with a lower risk of bleeding.^38^ Accordingly, coagulation abnormalities are not a contraindication *per se*; but, they should be considered as part of a broader risk assessment. Anatomical abnormalities, for example previous spinal surgery, severe scoliosis, morbid obesity, and difficulty in cooperation or positioning might make ESPB performance more challenging and outcomes less favourable, and can be viewed as relative contraindications.

## Complications

The ESPB is generally regarded as a safe technique. However, although complications have been anecdotally reported, their relative scarcity in the literature makes it difficult to accurately estimate their true incidence.

The most common complication is block failure or insufficient analgesia. Although some cases may be attributable to technical errors, inappropriate indications or suboptimal choice of volume or local anaesthetic concentration, as discussed previously, variability in block performance may also reflect characteristics of the technique itself, rather than representing an inherent limitation in all cases. Other possible complications include insertion site pain or discomfort and transient paraesthesia. Rare complications include haematoma and local infection, but very little has been reported in the literature with regard to these.^39^ There are several reports of motor weakness, with or without hypotension, presumably caused by epidural spread of local anaesthesic.^40^ Thus, clinicians should be cautious during insertion and when using continuous infusions. Similarly, pneumothorax is a potential risk, but there are no reliable reports in the literature.^41^

Several studies have reported that patients receiving continuous infusions have plasma local anaesthesia concentrations that exceed toxic thresholds.^42^ Although increased plasma concentrations in these patients rarely result in clinical signs of local anaesthetic systemic toxicity, cases of mild neurological symptoms and even seizures have been described.^42^ The addition of adrenaline (epinephrine) to the injectate may mitigate this risk;^43^ but, the risk of local anaesthetic systemic toxicity remains. When continuous erector spinae plane catheters are used, additional risks include catheter dislodgement, kinking, leakage and infection. While serious catheter-related infections appear rare, strict aseptic technique and regular catheter site inspection are recommended.^44^

Compared with paravertebral block, ESPB is associated with a lower incidence of block failure (0–2.3%), but a higher risk of haemodynamic instability, as well as increased risks of pneumothorax (0.4–3%) and vascular puncture (5–9%).^45^

## Conclusions

A decade after its introduction, the ESPB remains an attractive regional anaesthesia technique because of its technical simplicity, favourable safety profile and versatility across a broad range of Clinical scenarios. However, its mechanism of action is multifactorial and not entirely predictable. The ESPB should therefore be regarded as an effective component of a multimodal, opioid-sparing analgesic strategy rather than as a standalone technique, and its limitations must be clearly understood and acknowledged. Careful selection of patients, realistic expectations regarding dermatomal coverage and thoughtful integration with complementary analgesic modalities are essential to optimise outcomes.

## MCQs

The associated MCQs (to support CME/CPD activity) will be accessible at www.bjaed.org/cme/home by subscribers to BJA Education.

## Declaration of interests

SC has received honoraria from BBraun and Atricure for educational activities, fully reinvested in the UZ Leuven Funding for Fellows (F3) program to support locoregional anaesthesia fellows’ international education. KE or his institution have received funding from Fisher and Paykel Healthcare and GE Health Care. The other authors declare no conflicts of interest.
